# Helping behavior in prairie voles: A model of empathy and the importance of oxytocin

**DOI:** 10.1016/j.isci.2022.103991

**Published:** 2022-02-26

**Authors:** Kota Kitano, Atsuhito Yamagishi, Kengo Horie, Katsuhiko Nishimori, Nobuya Sato

**Affiliations:** 1 Department of Psychological Sciences, Kwansei Gakuin University, 1-1-155, Uegahara, Nishinomiya, Hyogo 662-8501, Japan; 2 Silvio O. Conte Center for Oxytocin and Social Cognition, Center for Translational Social Neuroscience, Yerkes National Primate Research Center, Emory University, 954 Gatewood Road, Atlanta, GA 30329, USA; 3 Department of Obesity and Inflammation Research, Fukushima Medical University, Fukushima, Fukushima 960-1295, Japan; 4 Department of Bioregulation and Pharmacological Medicine, Fukushima Medical University, Fukushima, Fukushima 960-1295, Japan

**Keywords:** Evolutionary biology, rodent behavior

## Abstract

Several studies suggest that rodents show empathic responses and helping behavior toward others. We examined whether prairie voles would help conspecifics who were soaked in water by opening a door to a safe area. Door-opening latency decreased as task sessions progressed. Female and male voles stayed close to the soaked voles' side at equal rates and opened the door with similar latencies. When the conspecific was not soaked in water, the door-opening latency did not decrease. This suggests that the distress of the conspecific is necessary for learning to open the door and that the door-opening performed by prairie voles corresponds to helping behavior. Additionally, we examined the helping behavior in prairie voles in which oxytocin receptors were genetically knocked out. Oxytocin receptor knockout voles demonstrated less learning of the door-opening behavior and less interest in soaked conspecifics. This suggests that oxytocin is important for the emergence of helping behavior.

## Highlights

• Prairie voles demonstrated helping behavior toward a cagemate in distress
• There was no difference in helping behavior depending on the helper’s sex
• Learning of the helping behavior was prevented when cagemates were not in distress
• Oxytocin receptor knockout prairie voles demonstrated less helping behavior

## Graphical abstract

**Figure:**
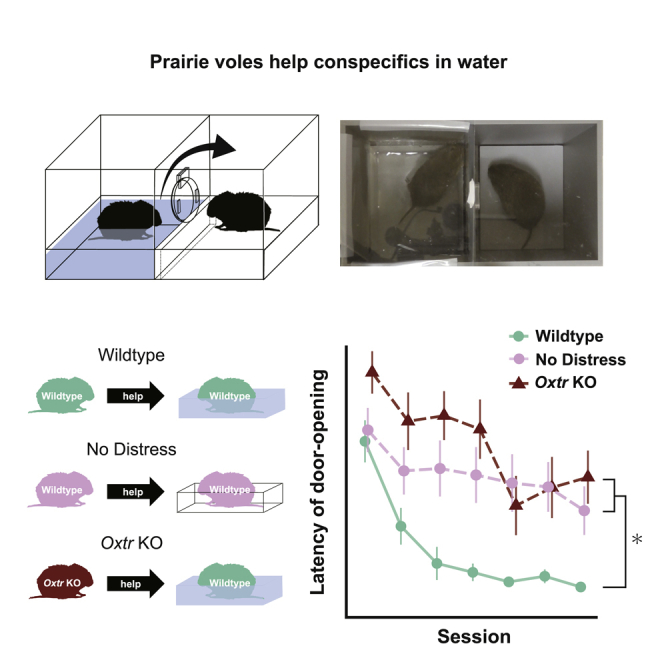

## Introduction

Empathy is an ability to experience and share the mental state of others (Decety et al., 2016; de Waal and Preston, 2017; Meyza et al., 2017; Preston and de Waal, 2002). The existence of empathy has been suggested in a variety of species, including non-human primates (Campbell and de Waal, 2011; Koski and Sterck, 2010; Pruetz, 2011), dogs (Palagi et al., 2015), birds (Gallup et al., 2015), and rodents (Bartal et al., 2011, 2016; Sato et al., 2015). In mammals, prosocial behaviors such as helping and consolation are essential for the development of society and are thought to be elicited by empathy (de Waal and Preston, 2017). Helping behavior, in which an actor pays a cost and provides a benefit to others, has been observed in animals that are considered to be highly intelligent, such as chimpanzees (Pruetz, 2011; Yamamoto et al., 2012), elephants (Schulte, 2000), and dolphins (Kuczaj et al., 2015). Recent studies suggest that rats also demonstrate helping behavior toward distressed conspecifics, such as those in a narrow tube (Bartal et al., 2011, 2016) and in water (Sato et al., 2015; Yamagishi et al., 2019, 2020). Detecting others' distress is a prerequisite for helping behavior (Cronin, 2012; Decety et al., 2016).

Prairie voles (*Microtus ochrogaster*) show various social behaviors, such as social bonding, nurturing, allogrooming, and huddling more often than other rodents such as mice and rats (Aragona and Wang, 2004; Carter and Getz, 1993; Getz and Carter, 1996; Getz and Hofmann, 1986). Because of this trait, many studies on social behavior have been carried out using prairie voles (reviewed by Aragona and Wang, 2004; Potretzke and Ryabinin, 2019; Tabbaa et al., 2017; Young and Wang, 2004). Previous studies suggest that prairie voles show empathy-like behavior, such as freezing caused by emotional contagion of conspecifics' fear, and consolation (Burkett et al., 2016; Stetzik et al., 2018; Wardwell et al., 2020). However, helping behavior that rescues conspecifics from distressed situations has not been elucidated in prairie voles. The purpose of this study was to investigate whether prairie voles display helping behavior using a door-opening paradigm, which has been used to examine helping behavior in rats (Bartal et al., 2011; Sato et al., 2015).

Several studies have reported that oxytocin and oxytocin receptors in rodents affect social behavior, including affiliative behavior, social cognitive behavior, and empathic responses (Bartz et al., 2010; Marlin and Froemke, 2017; Rogers-Carter et al., 2018; Ross and Young, 2009; Winslow and Insel, 2002; Young and Barrett, 2015). Especially in empathic responses, previous studies suggest that oxytocin has an effect across many species (Panksepp and Panksepp, 2013; Paradiso et al., 2021). In mice, intranasal oxytocin increases vicarious freezing in response to the fear of stranger mice (Pisansky et al., 2017). In monkeys, infusion of oxytocin increases preferences for performing actions to reward others (Chang et al., 2012). Such cross-species approaches can help clarify the evolutionary mechanisms of the effects of oxytocin on empathy (Decety et al., 2012). However, the role of oxytocin in helping behavior is largely unexamined except in studies that examined the effect of oxytocin administration and of blocking of oxytocin receptors in rats (Yamagishi et al., 2019, 2020).

In this study, we examined the effects of oxytocin on helping behavior using oxytocin receptor knockout (*Oxtr* −/−; *Oxtr* KO) voles. A genetic modification technique has been used to elucidate the function of the oxytocin system in social behavior (Boender and Young, 2020). Animals with genetically knocked out oxytocin function have been especially useful in such research (Froemke and Young, 2021). Studies with *Oxtr* KO mice have demonstrated abnormalities in social behavior, such as huddling, allogrooming, and nurturing (Pobbe et al., 2012; Rich et al., 2014). However, because of the restriction of social responses in mice (Beery et al., 2018; McGraw and Young, 2010), it is difficult to examine the details of oxytocin mechanisms involved in empathic behaviors such as helping behavior. In this study, we used prairie voles that demonstrate a variety of social behaviors to examine the function of oxytocin in helping behavior.

## Results

### Prairie voles demonstrate door-opening behavior regardless of the sex

**Figure 1:**
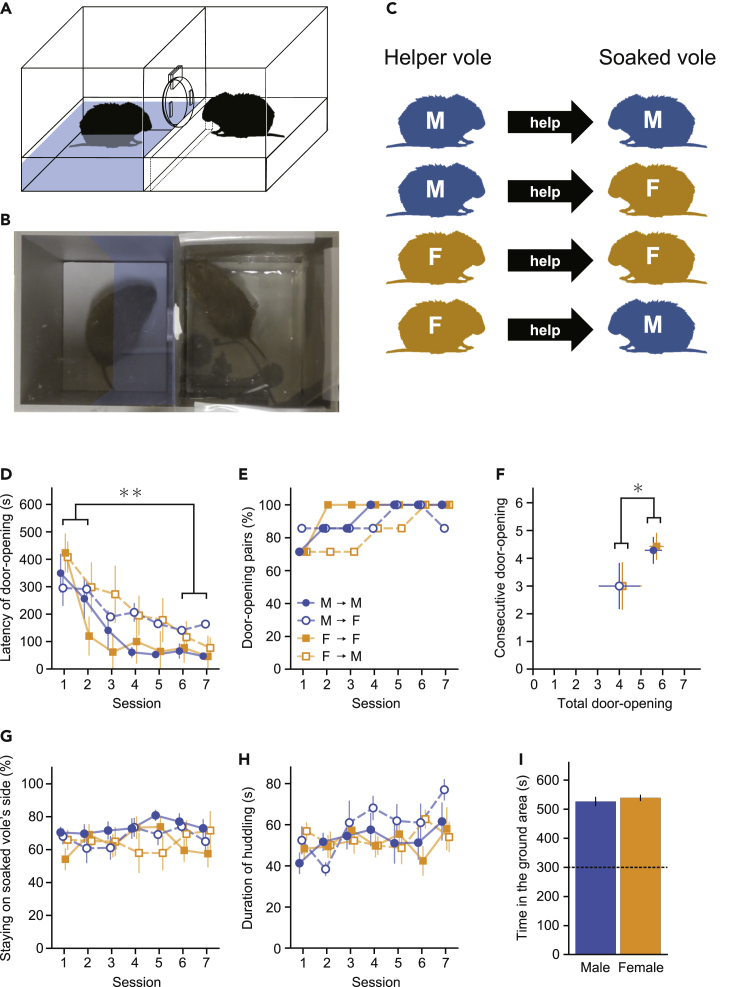
Experimental apparatus and behavioral results in wild-type voles (A) Schematic diagram of the experimental apparatus. A helper vole could rescue a soaked vole from the distressed situation of being in water by opening the circular door. (B) A photo image during the door-opening task. An acrylic plate covered both the ground and pool areas, which reflected the video camera and the ceiling of the laboratory. The area indicated by the blue shade is defined as the soaked vole’s side. (C) In this experiment, all sex combinations of the helper and soaked voles were examined. (D) The mean latency of door-opening in the helper voles of all sex combinations (n = 7 for all the groups; post hoc test using Holm’s method (∗∗ *p*s < 0.01), following the effect of session in a three-way ANOVA; *F*(6, 144) = 23.35, p < 0.001). (E) The percentage of door-opening pairs in each sex combination group. (F) The total number of door-opening sessions (two-way ANOVA; the effect of sex combination; *F*(1, 24) = 4.32, ∗p = 0.049) and the number of consecutive door-opening sessions. (G) The percentage of time that the helper stayed on the soaked vole’s side. (H) The mean duration of huddling during the 2-min interaction period after opening the door. (I) The time male and female helper voles spent in the ground area during the habituation period. The dashed line indicates the expected value. The error bars indicate the SE of means.

We examined door-opening behavior in prairie voles with a variety of paired sex combinations. Seven pairs of littermate voles with all sex combinations (7 pairs in each group derived from 4–7 litters) were tested using a door-opening task. One member of each pair was assigned to be a soaked vole, and the other was assigned to be a helper vole. The experimental apparatus consisted of a pool area and a ground area (Figures 1A and 1B). The helper vole in the ground area rescued the soaked vole in the pool area by opening a circular door (see Video S1). The helper vole could see the soaked vole through a transparent acrylic plate to which the circular door was attached. The task session started when the helper vole was placed in the ground area shortly after the soaked vole was placed in the pool area. We measured the latency of the door-opening from the placement of the helper vole in the ground area. After the helper vole opened the door within 10 min, the helper and soaked voles were allowed to interact for 2 min.

Video S1. An example scene of the Distress condition, related to Figure 1

The latency of door-opening decreased over the seven sessions in both the same-sex and opposite-sex pairs (Figures 1C, 1D, 1E, and S1). A three-way ANOVA for mixed design with between-subjects factors of sex combination (same-sex, opposite-sex) and helper’s sex (male, female), and a within-subject factor of session (7) revealed no main effect of sex combination (*F*(1, 24) = 2.51, p = 0.126) and helper’s sex (*F*(1, 24) < 0.01, p = 0.987). There was a significant effect of session (*F*(6, 144) = 23.35, p < 0.001). Post hoc tests using Holm’s method revealed that the door-opening latencies of sessions one and two were longer than those of sessions 6 and 7 (*p*s < 0.01). This suggests that the helper voles learned to open the door to free the soaked voles from the pool area regardless of the sex combinations of pairs. The interaction of sex combination × session was close to statistical significance (*F*(6, 144) = 1.88, p = 0.087). There were no other significant interactions (*p*s > 0.100). We also counted the total number of door-opening sessions and the number of consecutive door-opening sessions (Figure 1F). For the purpose of counting, the door-opening was defined as opening it within 200 s latency. A two-way ANOVA on the total number of door-opening sessions with between-subjects factors of sex combination and helper’s sex revealed a significant effect of sex combination (*F*(1, 24) = 4.32, p = 0.049). Neither a main effect of helper’s sex (*F*(1, 24) = 0.04, p = 0.852) nor one of sex combination × helper’s sex interaction (*F*(1, 24) < 0.01, p > 0.999) was observed. For the number of consecutive door-opening sessions, a two-way ANOVA revealed a close to significant effect of sex combination (*F*(1, 24) = 3.32, p = 0.081). Neither a main effect of helper’s sex (*F*(1, 24) = 0.01, p = 0.924) nor one of sex combination × helper’s sex interaction (*F*(1, 24) = 0.01, p = 0.924) was revealed. These results suggest that opposite-sex pairs may not demonstrate the door-opening behavior as well as same-sex pairs.

At the beginning of the experiment, i.e., before starting the sessions in which the voles learned the door-opening behavior, the helper voles experienced a habituation procedure in which they were placed in the ground area in the experimental apparatus for 10 min each day for two days. Because the circular door was opened and placed on the floor of the ground area during the habituation, the helper voles could go back and forth between the ground and pool areas. During the habituation period, the helper voles stayed longer in the ground area (532.77 ± 9.69 s, mean ± SD) than the pool area. The time spent in the ground area was significantly longer than the expected value (300 s, half of the habituation period, *t*(27) = 23.58, p < 0.001). In a comparison of the water aversion of male and female helper voles, a Welch’s t-test revealed no significant difference in the time spent in the ground area (*t*(22.57) = 0.63, p = 0.536; Figure 1I). This suggests that prairie voles have an aversion to water regardless of their sex.

To examine the degree of interest in the soaked cagemate expressed by the helper vole, we measured how long the helper vole stayed close to the soaked vole’s side in the ground area during the door-opening task. The ground area was divided into near and far halves relative to the pool area, and the time the helper vole spent in each half was measured (Figure 1G). A three-way ANOVA for mixed design with between-subjects factors of sex combination and helper’s sex, and a within-subject factor of session revealed neither an effect of sex combination (*F*(1, 24) = 2.11, p = 0.160), an effect of helper’s sex (*F*(1, 24) = 0.10, p = 0.756), nor an effect of session (*F*(6, 144) = 0.39, p = 0.885). There were no significant interactions either (*p*s > 0.100).

To examine social attachment, we measured the duration of huddling in the pairs of helper and soaked voles during the 2-min interaction period after the door was opened (Figure 1H). Huddling was defined as touching part of each other’s trunks. A three-way ANOVA for mixed design with between-subjects factors of sex combination and helper’s sex, and a within-subject factor of session revealed a main effect of session (*F*(6, 144) = 2.39, p = 0.031). Neither a main effect of sex combination (*F*(1, 24) = 0.95, p = 0.340) nor one of helper’s sex (*F*(1, 24) = 0.65, p = 0.428) was observed. There were no significant interactions (*p*s > 0.100).

### Door-opening behavior in prairie voles corresponds to helping behavior

**Figure 2:**
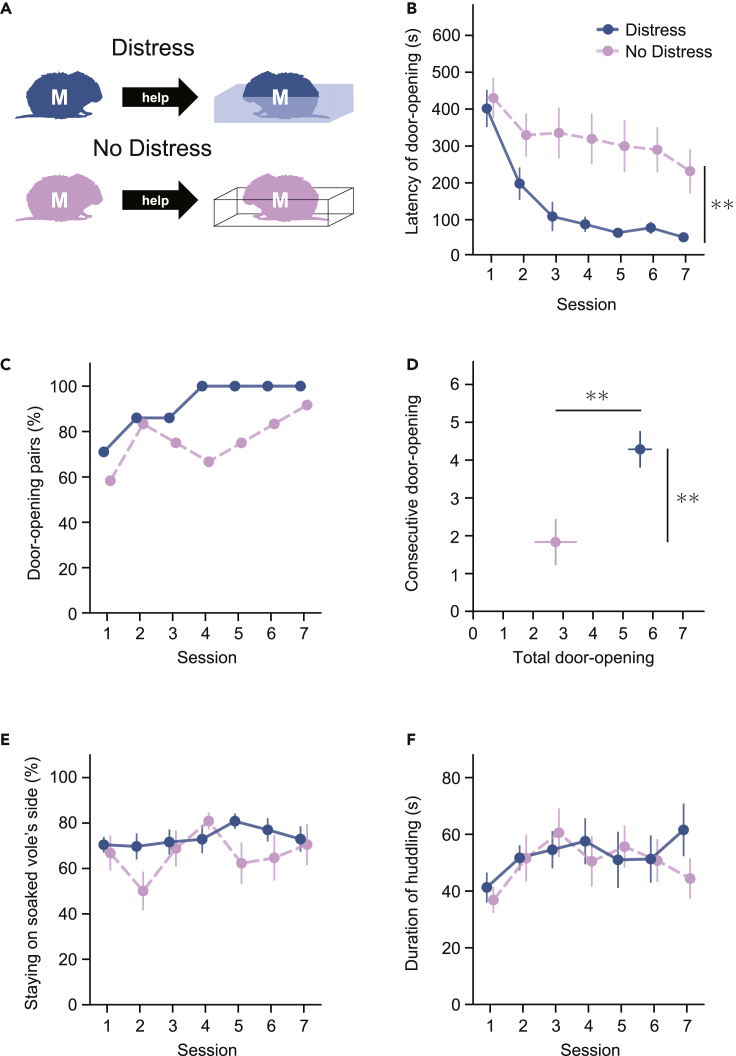
Prairie voles show prolonged latency of door-opening behavior toward non-soaked cagemates (A–C) There were two groups of paired voles. In one group, one of the pair (a cagemate of the helper vole) was soaked in water (Distress, seven pairs of same-sex males). In the other group, the cagemate was not soaked in water (No Distress, 12 pairs of same-sex males). The mean latency (B) and the percentage (C) of door-opening of the helper voles in the Distress and No Distress groups (two-way ANOVA; the effect of group; *F*(1, 17) = 5.98, ∗∗p = 0.003). (D) The total number of door-opening sessions (Welch’s t-test; *t*(16.26) = 3.30, ∗∗p = 0.004) and the number of consecutive door-opening sessions (Welch’s t-test; *t*(16.83) = 2.98, ∗∗p = 0.009) in the Distress and No Distress groups. (E) The percentage of time that the helper voles stayed on the soaked vole’s side in the Distress and No Distress groups. (F) The mean duration of huddling during the 2-min interaction period after opening the door in the Distress and No Distress groups. The error bars indicate the SE of means.

The door-opening behavior was potentially learned through factors other than empathy, for instance, social interaction. To examine the possibility that helping behavior is motivated by empathy, we tested the door-opening behavior of the helper voles when the cagemate was not soaked in water (Figure 2A; see Video S2). The helper voles (n = 12; all voles were in same-sex male pairs and were different from those used in the previous experiment) did not demonstrate a substantial decrease in the door-opening latency when their cagemate was on the ground instead of soaked in water (Figures 2B, 2C, and S2). We compared the door-opening latencies between when the cagemates were not soaked and when they were soaked (the aforementioned experiment: seven pairs of same-sex males). A two-way ANOVA for mixed design with a between-subjects factor of group (Distress; the cagemate was soaked in water, No Distress; the cagemate was not soaked in water) and a within-subject factor of session (7) revealed main effects of group (*F*(1, 17) = 5.98, p = 0.003) and session (*F*(6, 102) = 5.97, p < 0.001), while there was no significant group × session interaction (*F*(6, 102) = 1.17, p = 0.325). The total number of door-opening sessions (*t*(16.26) = 3.30, p = 0.004) and the number of consecutive door-opening sessions (*t*(16.83) = 2.98, p = 0.009) were larger in the Distress group than the No Distress group (Figure 2D). These results suggest that the learning is suppressed when the cagemates are not soaked in water, which in turn suggests that the negative emotions of the cagemate in the aversive situation are important for the helper voles' learning to open the door. Thus, the door-opening behavior is regarded as helping behavior.

Video S2. An example scene of the No Distress condition, related to Figure 2

We assessed the percentage of time that the helper voles stayed on the soaked vole’s side during the door-opening task (Figure 2E). A two-way ANOVA for mixed design with a between-subjects factor of group (Distress, No Distress) and a within-subject factor of session (7) revealed neither a main effect of group (*F*(1, 14) = 0.89, p = 0.362), session (*F*(6, 84) = 1.44, p = 0.208), nor a group × session interaction (*F*(6, 84) = 1.36, p = 0.239). This suggests that the helper voles' interest in the cagemates in the pool area is not influenced by whether they are soaked or not. In addition, we investigated the duration of huddling in the pairs of helper and soaked voles (Figure 2F). A two-way ANOVA revealed neither a main effect of group (*F*(1, 14) = 0.13, p = 0.729), session (*F*(6, 126) = 1.44, p = 0.208), nor a group × session interaction (*F*(6, 84) = 0.66, p = 0.681), suggesting that the huddling of prairie voles after the door is opened is not affected by whether the cagemate is soaked or not.

### Oxytocin receptor knockout voles demonstrate less helping behavior

**Figure 3:**
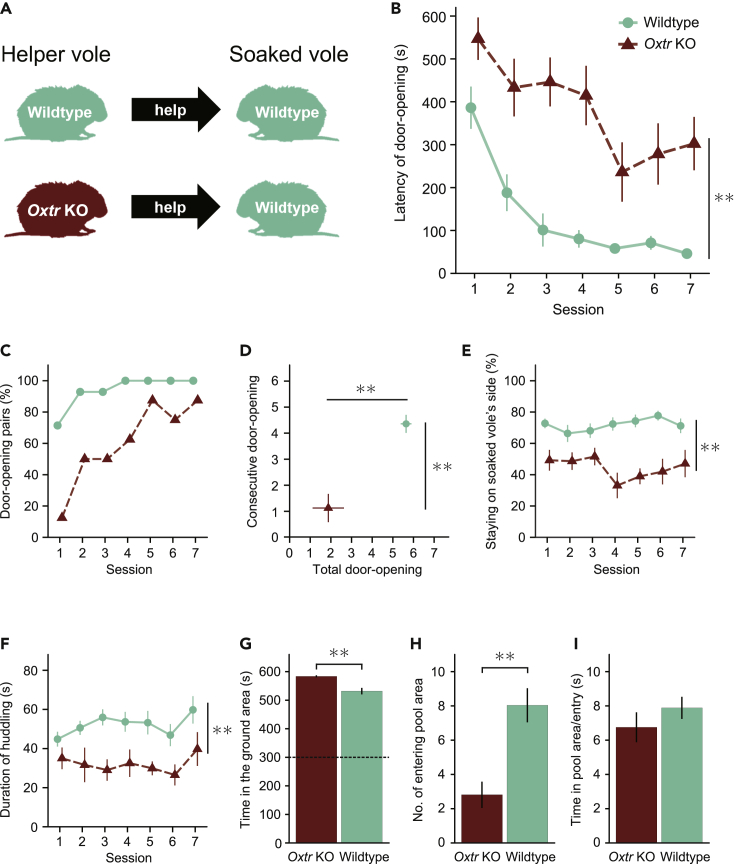
*Oxtr* KO voles demonstrate less helping behavior (A) In this experiment, the door-opening behavior of the helper voles with knocked out oxytocin receptors (*Oxtr* KO) was compared with that of wild type (*Oxtr* +/+). (B) The mean latency of door-opening in the helper voles of wild type (n = 14; seven were male and another seven were female; all were paired with a same-sex soaked cagemate) and the *Oxtr* KO helper voles (n = 8; four were male and another four were female; all were paired with a same-sex cagemate). The significant difference was tested with a two-way ANOVA (the effect of group; *F*(1, 20) = 27.93, ∗∗p < 0.001). (C) The percentage of door-opening pairs in the wild-type and *Oxtr* KO voles. (D) The total number of door-opening sessions (Welch’s t-test; *t*(8.57) = 4.41, ∗∗p = 0.002) and the number of consecutive door-opening sessions (Welch’s t-test; *t*(12.35) = 4.74, ∗∗p < 0.001). (E) The percentage of time that the helper voles stayed on the soaked vole’s side (two-way ANOVA; the effect of group, *F*(1, 20) = 41.87, ∗∗p < 0.001). (F) The mean duration of huddling during the 2-min interaction period after opening the door (two-way ANOVA; the effect of group; *F*(1, 20) = 9.49, *∗∗*p = 0.006). (G–I) The time spent in the ground area (G, Welch’s t-test; *t*(14.42) = 5.45, ∗∗p < 0.001), the number of entries into the pool area (H, Welch’s t-test; *t*(19.09) = 3.73, ∗∗p < 0.001), and the time spent in the pool area per entry (I) in the habituation period. The dashed line indicates the expected value. The error bars indicate the SE of means.

To investigate the impact of the elimination of oxytocin function on helping behavior, we examined the helping behavior in *Oxtr* KO voles and compared it with that in wild-type voles (n = 14, the same-sex male and female pairs in the aforementioned experiment). The *Oxtr* KO voles were homozygous for the knocked out oxytocin receptor gene. The *Oxtr* KO voles were paired with wild-type littermates (Figure 3A). All of the *Oxtr* KO voles were assigned to be helpers. The *Oxtr* KO helper voles and the soaked wild-type voles were in same-sex pairs (n = 8, four pairs for each sex).

The *Oxtr* KO voles did not demonstrate substantial reduction in the door-opening latency as compared with the wild-type voles (Figure 3B). Of the *Oxtr* KO voles, two did not open the door within the 10-min session even after the fourth session (Figures 3C and S3). The door-opening latency was compared between the *Oxtr* KO and wild-type voles using a two-way ANOVA for mixed design with a between-subjects factor of group (*Oxtr* KO, Wild type) and a within-subject factor of session (7). It revealed main effects of group (*F*(1, 20) = 27.93, p < 0.001) and session (*F*(6, 120) = 18.84, p < 0.001), while a group × session interaction was close to significant (*F*(6, 120) = 2.01, p = 0.070). Holm’s multiple comparisons of latencies in the *Oxtr* KO voles revealed no significant difference between all the sessions (*p*s > 0.05). Both the total number of door-opening sessions (*t*(8.57) = 4.41, p = 0.002) and the number of consecutive door-opening sessions (*t*(12.35) = 4.74, p < 0.001) were larger in the wild-type group than in the *Oxtr* KO group (Figure 3D). Next, we compared the time spent on the soaked vole’s side in the *Oxtr* KO voles with that in the wild-type voles (Figure 3E). A two-way ANOVA for mixed design revealed a main effect of group (*F*(1, 20) = 41.87, p < 0.001), but neither an effect of session (*F*(6, 120) = 0.57, p = 0.757) nor a group × session interaction (*F*(6, 120) = 1.55, p = 0.169). These results suggest that the *Oxtr* KO voles demonstrate less helping behavior and less interest in the soaked voles than the wild-type voles. Additionally, we examined the duration of huddling in the *Oxtr* KO voles (Figure 3F). A two-way ANOVA for mixed design revealed a main effect of group (*F*(1, 20) = 9.49, p = 0.006). Neither a main effect of session (*F*(6, 120) = 1.51, p = 0.181) nor a group × session interaction (*F*(6, 120) = 0.66, p = 0.685) was observed. This suggests that the lack of oxytocin reduces huddling behavior.

To evaluate the water aversion in the *Oxtr* KO voles, we compared the time spent in the ground and pool areas during the habituation period. The *Oxtr* KO voles stayed longer in the ground area (583.56 ± 3.53 s, mean ± SD) than the pool area, and the time spent in the ground area was significantly longer than the expected value (300 s, half of the habituation period, *t*(7) = 179.29, p < 0.001). In a comparison of the water aversion of the *Oxtr* KO and wild-type voles, a Welch’s t-test showed that the time spent in the ground area was longer in the *Oxtr* KO voles than in the wild-type voles (Figure 3G; *t*(14.42) = 5.45, p < 0.001). The number of entries into the pool area was significantly higher in the wild-type voles than in the *Oxtr* KO voles (Figure 3H; *t*(19.09) = 3.73, p < 0.001). No significant difference was found between the *Oxtr* KO and wild-type voles in time spent in the pool area per entry (Figure 3I; *t*(13.68) = 0.98, p = 0.342).

## Discussion

In this study, we investigated helping behavior in prairie voles using the door-opening paradigm. The results indicate that the helper voles quickly learned to open the door to free the soaked voles from water. In addition, the prairie voles demonstrated an aversion to water. This suggests that the helper voles rapidly demonstrate door-opening behavior when their cagemates are in an aversive situation. In addition, we found no sex difference in the latency of door-opening in the helper voles nor in their interest in the soaked vole, although the door-opening behavior tended to be less consistent and less prompt when releasing soaked voles of the opposite sex than those of the same sex. When the cagemates were not soaked in water, the helper voles did not immediately learn to open the door. This suggests that the cagemate being in a distressed situation is important for learning to open the door and that the door-opening corresponds to helping behavior. The *Oxtr* KO voles demonstrated less learning of the door-opening behavior and less interest in the soaked vole than the wild-type voles did. This suggests that oxytocin is important for the emergence of helping behavior.

Helping behavior has been observed in rats when conspecifics are in distressed situations, such as being trapped (Bartal et al., 2011) or soaked in water (Sato et al., 2015). In the present study, soaking in water was used as an aversive situation. In the habituation period, the helper voles stayed much longer in the ground area than in the pool area, even when they could go back and forth between the ground and pool areas. This suggests that prairie voles have an aversion to water and is consistent with previous studies that reported water aversion in rats (Morris, 1984; Sato et al., 2015). It has been explained that helping behavior is learned through emotional contagion, a form of empathy, which involves sharing the distress of others (Bartal et al., 2011; Decety et al., 2016; Sato et al., 2015). In the present study, the helper vole could see the soaked vole through a transparent acrylic plate. Perceiving the conspecifics' distress might have driven the helping behavior in the helper voles. When the helper voles observed the soaked voles in distress, they might have shared the soaked voles' distress through emotional contagion, and this may have motivated the helper voles to open the door (Sato et al., 2015).

When their cagemates were not soaked in water, the prairie voles were slower and less consistent in learning to open the door. There is a claim that in rats, social interaction instead of empathy motivates door-opening behavior (Silberberg et al., 2014), although this has been rebutted (Bartal et al., 2011; Cox and Reichel, 2019; Sato et al., 2015). In this study, when the cagemate was not soaked in water, the helper voles were substantially behind in learning to open the door. This situation in which the cagemate was not distressed did not induce empathy in the helper vole, while the helper and soaked voles were still able to socially interact after the door was opened. This implies that empathy for distressed others, rather than social interaction, is important for learning to open the door in the behavioral paradigm of the present study. Previous studies in rats have also shown that helping behavior is suppressed when others are not in distressed situations (Cox and Reichel, 2019; Sato et al., 2015). These studies suggest that empathy for the distress of others is more influential than social interaction as a trigger for door-opening behavior. In the present study, some of the helper voles in the No Distress condition opened the door with decreasing latency. This is concordant with one previous study (Cox and Reichel, 2019), but is not perfectly consistent with another (Sato et al., 2015) in which most rats did not open the door at all for a non-distressed cagemate. Helping behaviors occur even when the effects of social interaction are excluded (Cox and Reichel, 2019; Mason, 2021). However, that condition was not examined in the present study. To unveil helping behavior in prairie voles, further studies with well-controlled experiments will be needed.

There was no effect of the helper voles' sex on helping behavior in the present study. In rodents, prosocial behavior in females is more evident than in males (Langford et al., 2010; Schweinfurth et al., 2019). However, the effect of the helper’s sex on helping behavior in rats is controversial: A previous study reported that female rats helped a distressed conspecific more than male rats (Bartal et al., 2011), whereas another study reported no sex difference in helping behavior (Sato et al., 2015). Meanwhile, we also found that the helping behavior of the prairie voles toward a distressed conspecific of the opposite sex tended to be delayed as compared to that toward one of the same sex. In the present study, we examined helping behavior only toward littermates. Prairie voles demonstrate incest avoidance (McGuire and Getz, 1981). Although the details are issues for future research, such an avoidance tendency might have caused the delay in the appearance of helping behavior.

The latency of door-opening in the *Oxtr* KO voles decreased less than that in the wild-type voles, suggesting that blocking the function of oxytocin has a significant negative impact on helping behavior. Many studies have shown that oxytocin and oxytocin receptors are involved in a variety of social behaviors in humans (Kosfeld et al., 2005; Zink and Meyer-Lindenberg, 2012), non-human primates (Ferguson et al., 2000; Smith et al., 2010), and rodents (Amico et al., 2004; Anacker and Beery, 2013; Pobbe et al., 2012; Rich et al., 2014). In particular, studies in mice indicated that oxytocin gene knockout caused deficits in social recognition (Bielsky and Young, 2004; Ferguson et al., 2001), in formation of social bonds (Liu and Wang, 2003; Young and Wang, 2004), and in social rewards (Dölen et al., 2013; Hung et al., 2017). High sociality in prairie voles may be related to a higher density of oxytocin receptors in the brain as compared to close relative species (Olazábal and Young, 2006; Ross et al., 2009). A previous study reported that oxytocin receptor knockout voles demonstrated autism-like behavior such as a lack of interest in social novelty (Horie et al., 2019). Similarly, the *Oxtr* KO voles in the present study demonstrated less interest in their cagemate. Nurturing and social play behavior in oxytocin knockout or oxytocin receptor knockout rodents as well as wild-type rodents is influenced by social context (Bredewold et al., 2014) and sexuality (Nishimori et al., 1996; Young et al., 1996; Zimmermann-Peruzatto et al., 2017). Helping behavior in oxytocin knockout voles may also be affected by social context and sexual interaction. Future studies will examine the interaction between oxytocin receptor knockout and these social elements.

We used huddling as an index for social attachment in the present study in accordance with a previous report (Barrett et al., 2015). A negative effect of blocking the oxytocin function on huddling suggests that oxytocin is involved in social attachment. However, huddling in prairie voles has generally been explored in the context of a partner preference test (Walum and Young, 2018). In the present study, it was measured in the interaction period just after the voles were exposed to the negative situation in which one of them was soaked in water. The decrease in huddling in the *Oxtr* KO voles might reflect interference with the social buffering that compensates for anxiety (Burkett et al., 2016) or simply an increase in sensitivity to the negative emotion itself (Amico et al., 2004).

Oxytocin has been gaining attention for its effects on social cognition in recent years (Bosch and Neumann, 2012; Decety et al., 2016; Insel, 2010; King et al., 2016; Rodrigues et al., 2009; Young and Wang, 2004). Specifically, several studies have reported that intranasal administration of oxytocin can alleviate some symptoms of autism (Guastella et al., 2010; Hollander et al., 2003; Preti et al., 2014). The present study implies a relationship between helping behavior and oxytocin in prairie voles. Further studies will be needed to manifest the details, and they may shed light on the mechanisms of social cognition and related psychological disorders. Experiments in prairie voles will be beneficial for examining social cognitive functions.

### Limitations of the study

The helping behavior learning deficits in the *Oxtr* KO voles suggest that blocking of oxytocin function disturbs empathic processes. However, such a learning disturbance could be caused by other effects. First of all, the blocking of oxytocin function might increase anxiety about water. In the *Oxtr* KO voles, the time spent in the pool area was shorter and the frequency of entering the pool area was lower than in the wild-type voles. This might have prevented the *Oxtr* KO voles from approaching the pool area, thus preventing the helping behavior. However, the profile of the door-opening latencies in the *Oxtr* KO voles (Figure 3B) was similar to that in the wild-type voles in the No Distress condition (Figure 2B), and there was no significant difference between them (*F*(1, 18) = 1.00, p = 0.331). In addition, stronger water aversion might have enhanced empathy for the soaked vole. In rats and mice, the emotional contagion of others' distress brought about by foot shocks increased if the rats and mice had experienced the foot shocks in the past (Atsak et al., 2011; Sanders et al., 2013). Because there was also a difference in the duration of huddling, it would be appropriate to interpret the reduced approaching of the soaked vole’s side in the *Oxtr* KO voles as being due to the effect of blocked oxytocin function on social factors rather than on water aversion. The second possible effect of the blocking of oxytocin function other than that on empathy is reduction of the motivation to engage in social contact. In the present study, however, the door-opening behavior in the *Oxtr* KO voles was not examined with the No Distress condition in which the cagemate is not soaked in water. This issue should be examined in the future. Finally, the *Oxtr* KO vole might have been in a hypoactive state. In the habituation period, there was a difference in the number of times the *Oxtr* KO and wild-type voles entered the pool area, whereas there was no difference in the time spent in the pool area per entry. This may suggest that the wild-type voles are generally more active than the *Oxtr* KO voles. However, in a previous study, there was no significant difference in activity level in an open field test between *Oxtr* KO and wild-type voles (Horie et al., 2019). Further studies are needed to exclude these explanations for the effect of blocking oxytocin functions. In addition, in the present study, we compared helping behavior in the *Oxtr* KO voles with that in wild-type voles with a different lineage. It should have been compared with wild-type or heterozygote littermates of the *Oxtr* KO voles. In future studies, using such a proper control group will be desirable.

All of the helper and soaked voles in the present study were littermates. Various factors of social context are involved in empathy (Bartal et al., 2014; Yamagishi et al., 2019). A previous study examining the emotional contagion of pain in mice suggests that the pain of familiar individuals is more contagious than that of strangers (Langford et al., 2006). Prairie voles exhibit allogrooming that functions as social buffering to relieve the fear of their partner. Allogrooming to a cagemate and a sibling is observed more frequently than that to a stranger (Burkett et al., 2016). The fact that the helper and soaked voles were littermates in the present study might have facilitated the helper voles' learning to release the soaked voles more quickly than if they were strangers to each other. Rats display as much helping behavior toward strangers as toward cagemates (Bartal et al., 2014; Yamagishi et al., 2019). It is unclear whether prairie voles exhibit helping behavior toward strangers. The effects of familiarity and kinship on helping behavior in prairie voles should be investigated in the future.

## STAR★Methods

### Key resources table

**Table:** 

REAGENT or RESOURCE	SOURCE	IDENTIFIER
**Experimental models: Organisms/strains**		

Wildtype and *Oxtr* KO prairie voles	Kwansei Gakuin University	N/A

**Oligonucleotides**		

Primer: *Oxtr*_F 5′-AGA TCA GTG CCC GGG GGG TGC CC-3′	FASMAC	N/A
Primer: *Oxtr*_R 5′-TCG AGC GAC ATA AGC AGC AG-3′	FASMAC	N/A

**Recombinant DNA**		

*Oxtr* KO prairie voles	Horie et al. (2019)	Gene ID: 101979991

**Software and algorithms**		

Excel	Microsoft Office	https://www.microsoft.com/
R	R foundation	https://www.r-project.org/
RStudio	R Studio	https://www.rstudio.com/

### Resource availability

#### Lead contact

Further requests should be directed to and will be fulfilled by the Lead contact: Nobuya Sato (nsato@kwansei.ac.jp).

#### Materials availability

The study did not generate any unique reagent.

### Experimental model and subject details

#### Subjects

We used 80 experimentally naïve wildtype (52 males and 28 females) and 16 naïve oxytocin receptor knockout (*Oxtr* KO, 8 males and 8 females) prairie voles (*Microtus ochrogaster*) maintained in a vivarium at Kwansei Gakuin University. The wildtype voles were kindly supplied to Tohoku University, from colonies maintained by Prof. Larry J. Young, who captured original source animals in Illinois, USA (Keebaugh et al., 2015). After the genetic manipulation of voles, performed at Tohoku University (v4 strain of Horie et al., 2019), the wildtype and *Oxtr* KO voles were transferred to and bred at Kwansei Gakuin University. At the beginning of the experiments, the mean age of the prairie voles was 21.1 weeks (range: 10–62 weeks) and the mean weight was 38.3 g (range: 25–63 g). They were housed in plastic home cages (320 × 212 × 130 mm, or 425 × 265 × 155 mm) with paper-chip bedding. The average number of prairie voles per cage was 3.9 (range: 2–6). Prairie voles in the same cage were littermates and were individually marked by ear punches. Helper and soaked voles were randomly assigned to individuals in the same cage. All animals were allowed free access to food (Labo MR Stock, NOSAN, Japan) and water. The vivarium was maintained at a constant temperature of 22°C and 60% humidity. The light-dark cycle was set to 12:12 h with lights on at 9:00 am. This experiment was approved by the Animal Experimentation Committee of Kwansei Gakuin University (2018-44, 2019-06).

#### Genotyping

Genotyping to discriminate between wildtype (*Oxtr* +/+) and *Oxtr* KO (*Oxtr* –/–) voles was done using the method of Horie et al. (2019). To summarize, we collected samples from prairie voles' ear tissues and used them for a polymerase chain reaction. In the polymerase chain reaction, the forward polymer was 5′-AGA TCA GTG CCC GGG GGG TGC CC-3′ and the reverse polymer was 5′-TCG AGC GAC ATA AGC AGC AG-3′. The prairie voles that were homozygous for the knocked out oxytocin receptor gene (*Oxtr*–/–) and those that were wildtype (*Oxtr* +/+) were used as the helper and soaked voles in the experiment, respectively.

### Method details

#### Experimental setup

The experimental apparatus was similar to that used in Sato et al. (2015). The size of the experimental box was smaller (240 × 120 × 210 mm) and it was made of gray polyvinyl chloride boards (5 mm in thickness). It consisted of ground and pool areas separated by a transparent acrylic plate (3 mm in thickness). During the experiment, the pool area contained water with a depth of 25 mm to create an aversive situation for the prairie voles. The transparent acrylic plate had a circular hole (51 mm in diameter). On the ground area side, the hole was covered with a circular door (60 mm in diameter, 3 mm in thickness). The door completely separated the ground area from the pool area. A helper vole in the ground area opened the door, allowing a soaked vole to escape from the pool area to the ground area. The difficulty of opening the circular door was set to a constant level by holding it between two transparent acrylic fragments (Figure 1A). One pushed up the circular door with springs from below and the other was attached to the transparent acrylic plate. To prevent soaked voles from interfering with the circular door, a thin transparent sheet was put on the surface of the pool area side of the transparent acrylic plate. The transparent sheet had three small holes in the area that overlapped the circular door. After the start of the experiment, two thin transparent plates (150 × 150 mm, 5 mm in thickness) were placed on top of the experimental apparatus to prevent the voles from escaping out of it. The experiment was recorded by a video camera (HDR-CX590, Sony) mounted above the experimental apparatus.

#### Habituation

Before the experiment, we habituated the helper voles to the experimental apparatus. The habituation was performed for 10 minutes per trial for two days for each helper vole. During the habituation, the door was detached from the dividing plate and was laid on the floor of the ground area. The helper voles could move freely between the ground and pool areas during the habituation. The pool area was filled with water.

#### Task procedure

Before the door-opening task, the experimental apparatus was cleaned with a 20% alcohol solution, the circular door was placed on the plate, and water was poured into the pool area to a depth of 25 mm. Immediately after the soaked vole was placed in the pool area, the helper vole was placed in the ground area. We measured the latency of the door-opening from the placement of the helper vole in the ground area. A trial of the task was carried out for a maximum of 10 minutes. If the helper vole opened the door in 10 minutes, we pulled out the sheet attached on the pool side of the plate to allow the soaked vole to escape to the ground area and the two voles to interact with each other. The duration of the interaction was two minutes. If the helper vole did not open the door within 10 minutes, the experimenter slightly opened the door to the right side and continued the trial for five more minutes. When the door-opening behavior was observed during those five minutes, the transparent sheet was removed to allow for interaction. If the helper vole did not open the door in the extra five minutes, the experimenter opened the circular door completely and removed the sheet to allow the interaction. This door-opening task was carried out in one trial per day, for a total of seven days.

### Quantification and statistical analysis

We analyzed the latency of door-opening and the time that the helper vole stayed on the soaked vole's side during the first 10 minutes of the task. The soaked vole's side was defined as the pool area side of the ground area divided in half. If the helper vole did not open the door within 10 minutes, the latency of door-opening of the trial was recorded as 600 s. We also measured whether the helper and soaked voles displayed huddling in the two-minute interaction period after the door-opening. Huddling was defined as touching parts of each other’s trunks. During the habituation of the helper voles, we measured the time that the helper voles stayed in the ground and pool areas. For the statistical analyses, we performed ANOVAs. Post-hoc tests were performed by multiple comparisons using Holm’s method. For the statistical analysis of water aversion, the total number of door-opening sessions and the number of consecutive door-opening sessions in Figures 2 and 3, we performed Welch’s t-tests. RStudio (version 1.1.453) was used for all statistical analyses. Significance values are reported as follows: ∗ *p* < .05 and ∗∗ *p* < .01. For the figures, all data are given as the mean ± standard error. Detail information of the statistic test is embedded in figure legends.

## Data Availability

• The raw datasets for figures in the paper are available at Mendeley data (https://doi.org/10.17632/h5m4tj9dzv.1). • This paper does not report original code. • Any additional information required to reanalyze the data reported in this paper is available from the lead contact upon request.
