# Supplementary material for: Helping behavior in prairie voles: A model of empathy and the importance of oxytocin
Source: iScience. 2022 Feb 26;25(4):103991. doi: 10.1016/j.isci.2022.103991 (PMC8931361; doi:10.1016/j.isci.2022.103991)
Supplement: Document S1. Figures S1–S3 [file mmc1.pdf]

iScience, Volume *25*

Supplemental information

Helping behavior in prairie voles: A model of empathy and the importance of oxytocin

Kota Kitano, Atsuhito Yamagishi, Kengo Horie, Katsuhiko Nishimori, and Nobuya Sato

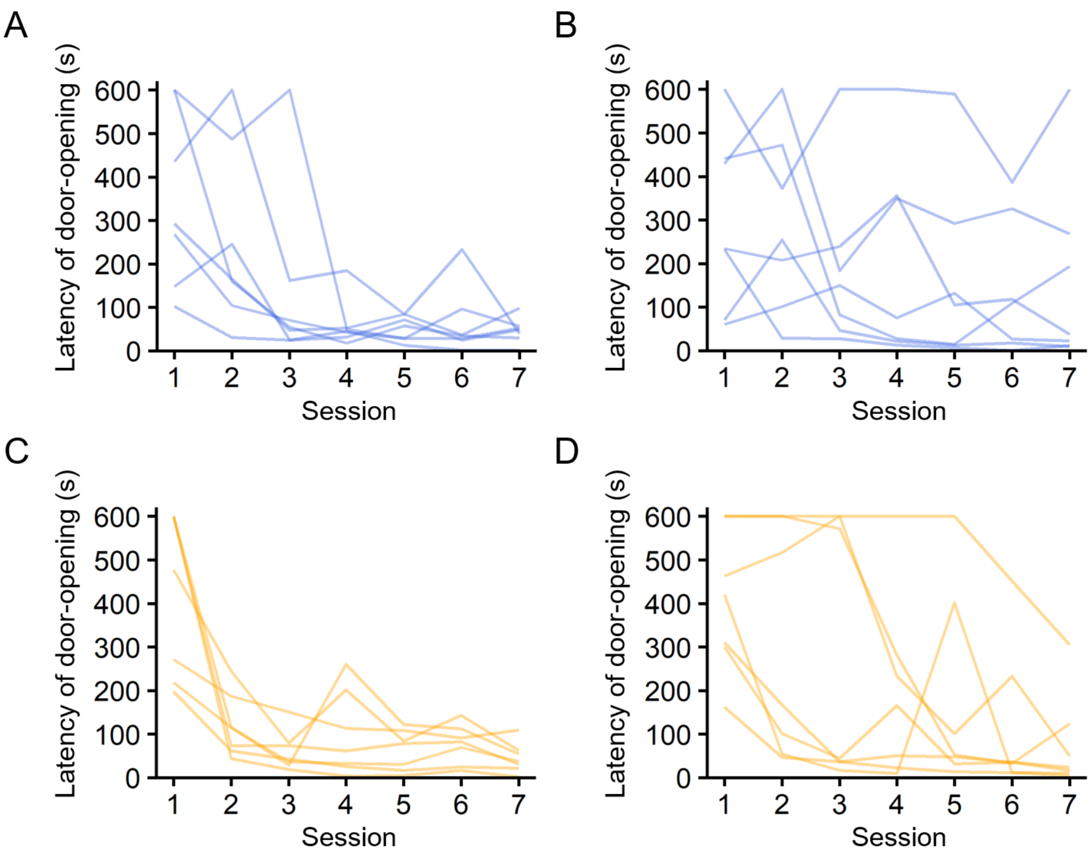

Figure S1. Individual data of the helper voles in all sex combinations, related to Figure 1.

Individual latency of door-opening in all sex combinations of pairs: a male helping another male (A), a male helping a female (B), a female helping another female (C), a female helping a male (D).

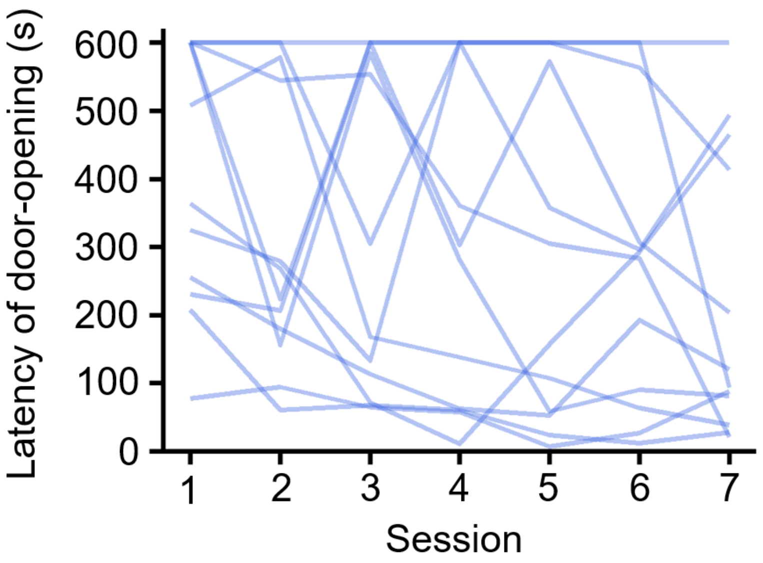

Figure S2. Individual data of the helper voles when the cagemates were not soaked in water, related to Figure 2.

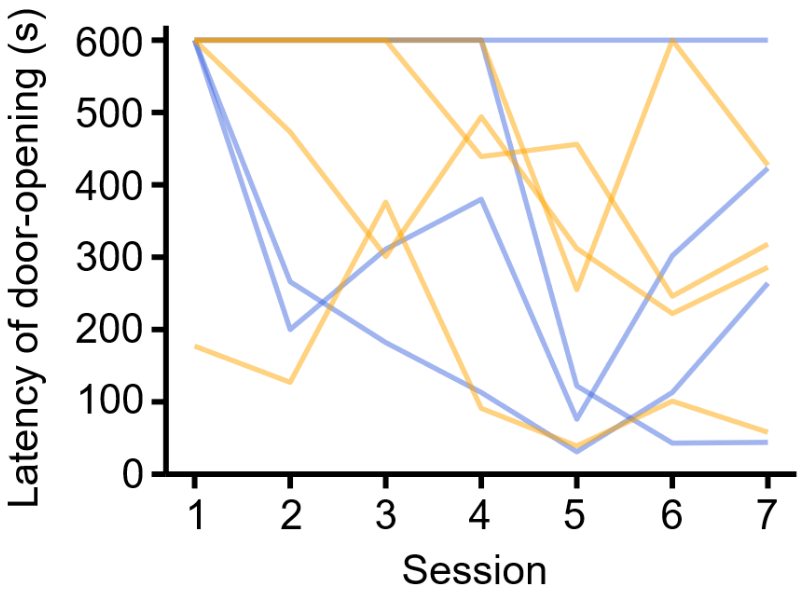

Figure S3. Individual data of the helpers of the *Oxtr* KO voles, related to Figure 3.

The blue and orange lines indicate the data of the male and female helper voles.
